# Identification and Functional Characterization of Adenosine Deaminase in *Mucor circinelloides*: A Novel Potential Regulator of Nitrogen Utilization and Lipid Biosynthesis

**DOI:** 10.3390/jof8080774

**Published:** 2022-07-26

**Authors:** Shaoqi Li, Junhuan Yang, Hassan Mohamed, Xiuwen Wang, Shuxian Pang, Chen Wu, Sergio López-García, Yuanda Song

**Affiliations:** 1 Colin Ratledge Center for Microbial Lipids, School of Agricultural Engineering and Food Science, Shandong University of Technology, Zibo 255000, China; lsq163947@126.com (S.L.); hassanmohamed85@azhar.edu.eg (H.M.); wangxiuwen2018@163.com (X.W.); pangshuxianxian@126.com (S.P.); wuchenjiayou1@163.com (C.W.); 2 Department of Food Sciences, College of Food Science and Engineering, Lingnan Normal University, Zhanjiang 524048, China; judywoniu@163.com; 3 Department of Botany and Microbiology, Faculty of Science, Al-Azhar University, Assiut 71524, Egypt; 4 Department of Genetics and Microbiology (Associated Unit to IQFR-CSIC), Faculty of Biology, University of Murcia, 3100 Murcia, Spain; slg5@um.es

**Keywords:** adenosine deaminase, *Mucor circinelloides*, lipid accumulation, nitrogen metabolism

## Abstract

Adenosine deaminase (ADA) is an enzyme distributed in a wide variety of organisms that cleaves adenosine into inosine. Since inosine plays an important role in nitrogen metabolism, ADA may have a critical function in the regulation of fatty acid synthesis. However, the role of ADA in oleaginous fungi has not been reported so far. Therefore, in this study, we identified one *ada* gene encoding ADA (with ID scaffold0027.9) in the high lipid-producing fungus, *Mucor circinelloides* WJ11, and investigated its role in cell growth, lipid production, and nitrogen metabolism by overexpressing and knockout of this gene. The results showed that knockout of the *ada* altered the efficiency of nitrogen consumption, which led to a 20% increment in the lipid content (25% of cell dry weight) of the engineered strain, while overexpression of the *ada* showed no significant differences compared with the control strain at the final growth stage; however, interestingly, it increased lipid accumulation at the early growth stage. Additionally, transcriptional analysis was conducted by RT-qPCR and our findings indicated that the deletion of *ada* activated the committed steps of lipid biosynthesis involved in acetyl-CoA carboxylase (*acc1* gene), cytosolic malic acid enzyme (*cme1* gene), and fatty acid synthases (*fas1* gene), while it suppressed the expression of AMP-activated protein kinase (*ampk α1* and *ampk β* genes), which plays a role in lipolysis, whereas the *ada*-overexpressed strain displayed reverse trends. Conclusively, this work unraveled a novel role of ADA in governing lipid biosynthesis and nitrogen metabolism in the oleaginous fungus, *M. circinelloides*.

## 1. Introduction

Microbial lipids, as environmentally sustainable and economically viable alternative sources of unusual lipids and fuels [1,2], have obtained increased attention since they have been used in the designing of high-value lipid products, particularly polyunsaturated fatty acids (PUFAs) such as alpha-linolenic acid (ALA), gamma-linolenic acid (GLA), and stearidonic acid (SDA), and others which have a variety of applications (i.e., in cosmetics, food, and healthcare industries) [3,4]. It is now possible to optimize the microbial lipid synthesis pathway to meet increasing market demands through metabolic engineering in microorganisms [5].

Nowadays, in order to produce a large number of oleochemicals, genetic modifications are performed in the varied microorganisms that are able to accumulate large quantities of lipids [6]. An oleaginous fungus, *Mucor circinelloides,* has been widely used for microbial lipid and lipid derivative production since it can accumulate lipids as nearly 36% of its cell dry weight (CDW); meanwhile, this fungus has efficient genetic engineering tools and a clear and known genetic background [7,8]. Moreover, its metabolism, specifically the production of the most diverse fatty acids, has been extensively studied and characterized [8,9]. Previous studies showed that a cascade of biochemical events has been triggered by nitrogen deficiency, which disturbed the TCA cycle and redirected carbon flux to lipid biosynthesis in *M. circinelloides* [10,11,12]. As postulated by Ratledge, lipid synthesis in *M. circinelloides* was regulated by a disparate number of enzymes, such as malic enzyme (ME), ATP-citrate lyase (ACL), acetyl-CoA carboxylase (ACC), and fatty acid synthase (FAS), which have been identified as conditional levers for fatty acid synthesis [11,13,14]. Many efforts, such as increasing the supply of precursors and diverting the carbon flux to TAGs, were made to increase the lipid production by changing the expression levels of the key enzymes involved in lipid synthesis in *M. circinelloides* [15,16,17]. Therefore, the genetic modification of the key enzymes involved in lipid synthesis was one of the celebrated strategies developed for achieving the profitable production of lipids in oleaginous fungi [18]. 

Adenosine deaminase (ADA; EC 3.5.4.4), as a crucial deaminating enzyme, catalyzes the irreversible conversions of adenosine to inosine and 2′-deoxyadenosine to 2′-deoxyinosine [19]. Meanwhile, it was ubiquitously expressed and involved in many cellular processes, including the biosynthesis and regulation of nucleic acids [20]. The ADA purified from *Aspergillus oryzae* has been found to catalyze the deamination of free adenine, adenine nucleotides, and oligonucleotides [21]. In addition, Lin et al. constructed adenosine deaminase-deficient recombinants to block the transformation of adenosine into hypoxanthine and enhance glutathione production in *Escherichia coli* and *Saccharomyces cerevisiae* [22]. Previous studies have also demonstrated that ADA is a possible producer of reactive oxygen species (ROS), which cause lipid peroxidation in cells and have a negative correlation with antioxidant enzymes [23,24]. In addition, increasing evidence has highlighted that ADA is associated with increased levels of hemoglobin (HbA1c), which plays a critical role in the derangement of lipogenesis in diabetes [25,26]. These studies showed that there is a connection between lipid metabolism and adenosine deaminase; however, research has rarely worked on the association of lipid metabolism with ADA, especially in oleaginous microorganisms.

Therefore, the present study aimed to investigate the role of ADA in the regulation of lipid biosynthesis in *M. circinelloides*. We constructed the *ada* gene knockout and overexpressing strains and studied their growth patterns, and the lipid accumulation in the cells. Additionally, we measured the mRNA expression levels of fatty acid metabolism-related genes in *M. circinelloides* to investigate the interconnection between ADA and lipid biosynthesis. To the best of our knowledge, this study provides the first report exploring the role of ADA in lipid accumulation and nitrogen metabolism in *M. circinelloides* WJ11.

## 2. Results

### 2.1. Identification of Gene Coding for the ADA in M. circinelloides

Based on the known completed genomic sequencing of *M. circinelloides* WJ11, a potential gene of adenosine deaminase ADA (namely *ada* encoded by scaffold0027.9) was identified by the genome-wide analysis. To further evaluate the general properties of the *ada* gene, the bioinformatical analysis of the genes was carried out by online website tools including ProtParam, CFSSP, and SOPMA. The ADA protein consists of 338 amino acids and exhibits good stability in water (the instability index is 38.79). Analysis of the grand average of hydropathicity (−0.250) indicated that ADA is hydrophilic. In addition, the ADA_AMPD domain (cd00443) was observed in ADA through CDD blast in the conserved domain database (NCBI-CDD), which suggested that this protein may have a catalytic role in adenosine deamination.

**Figure 1:**
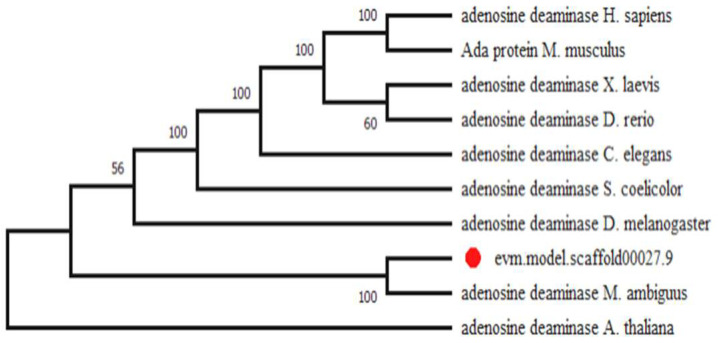
Phylogenetic analysis of ADA using MEGA6. The ADA from the model organism was identified by BLASTp and aligned using the neighbor-joining method. The sequences are *Homo sapiens* (NP_000013.2), *Mus musculus* (AAH02075.1), *Xenopus laevis* (NP_001085740.1), *Danio rerio* (AAH76532.1), *Caenorhabditis elegans* (NP_872091.1), *Streptomyces coelicolor* (CAC33066.1), *Drosophila melanogaster* (NP_649866.1), *M. circinelloides*, *Mucor ambiguus* (GAN00815.1), and *Arabidopsis thaliana* (NP_192397.2).

Phylogenetic evolution analysis was employed to explore the evolutionary relationship of the ADA in *M. circinelloides* WJ11 and its homologous proteins from other species. As shown in Figure 1, ADA protein occurs ubiquitously in animals, plants, and eukaryotic microorganisms. The ADA of *M. circinelloides* WJ11 shared 89% sequence similarities with GAN00815.1 of *Mucor ambiguus*, about 21% with AAH76532.1 of *Danio rerio*, and 23% with NP_000013.2 of *Homo sapiens*. Analysis of the amino acid sequences by NCBI-CDS exhibited that they all contain the conserved domain of the metallo-dependent hydrolase superfamily (cl00281), which has a conserved metal-binding site deprotonating a water molecule for a nucleophilic attack on the substrate in the common reaction mechanism. Similar to the tertiary structure of mouse ADA, the predicted three-dimensional structure of the ADA in *M. circinelloides* displayed an α/β-barrel structure with a zinc atom within the active site, which can catalyze the deamination of adenosine (Figure S1) [27]. Meanwhile, ADA has been observed in all human tissues in three isoforms: ADA1, ADA1+CP, and ADA2, involved in extracellular adenosine degradation [28]. Furthermore, an extensive search of the *Danio rerio* genome, followed by a phylogenetic analysis, confirmed the presence of three distinct members of ADA (ADA1, ADA2, and ADAL), which may take part in a complex regulation of adenosine deaminase activity [29]. In summary, these findings signified that the putative ADA could be responsible for adenosine degradation in *M. circinelloides*.

### 2.2. Generation of Ada Overexpressing and Knockout Transformants of M. circinelloides

**Figure 2:**
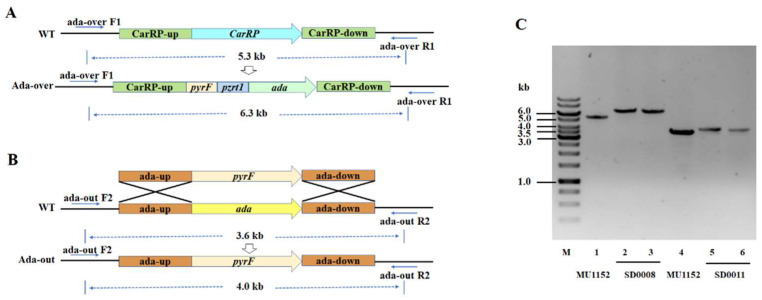
Overexpression and knockout of *ada* gene. (**A**) The structure of *ada*-overexpressing plasmid, pCRC8. (**B**) Genomic structure of *ada* wild-type locus (middle) and upon homologous recombination (lower) with the replacement fragment (upper). (**C**) PCR amplification of the plasmid region in MU1152 (WT), and *ada*-overexpressing and *ada*-knockout strains, SD0008 and SD0011.

The *ada* overexpressing and knockout mutants of *M. circinelloides* were generated to investigate the effect of ADA on lipid biosynthesis in *M. circinelloides.* The gene *ada* was overexpressed using the plasmid pMAT2075, which contained the *pyrF* gene as the selectable marker and a promoter *zrt1*, flanked by the sequences corresponding to regions surrounding the carotenogenic *carRP* gene (Figure 2A). Then, the gene overexpressing fragment was cut by *Sma I* and transformed into MU760, which is the leucine and uracil auxotroph of WJ11, by electroporation. The selection of homokaryotic transformants was conducted using the methods described by Rodríguez-Frómeta et al. [30]. The transformant was checked by PCR amplification with the relevant primers, ada-over-F1/R1, which obtained a 6.3-kb fragment from the *ada*-overexpressed strain, while a 5.3-kb fragment was obtained from the control strain (Figure 2C). The knockout plasmid was designed by a gene replacement strategy, using the *pyrF* gene as the selective marker and flanking the adjacent sequences of the *ada* gene to allow homologous recombination (Figure 2B). The fragments obtained from these designed vectors were cut by *Not I* and transformed into MU760. The construction of the *ada*-knockout mutant was confirmed by PCR amplification with the ada-out-F2/R2 primers (Figure 2C). The amplification results showed an expected 4.0-kb band in the *ada*-knockout strain and a 3.6-kb band in the control, respectively. Consequently, the above PCR analysis validated the target gene had been integrated into the genome of the overexpressed transformant and that was named as SD0008. Meanwhile, the absence of the 3.6-kb wild-type fragment indicated that the *ada* wide-type allele had been replaced and the transformant was named as SD0011, which was the homokaryon for the *ada* knockout.

### 2.3. Analysis of Ada Expression in the Transformants

**Figure 3:**
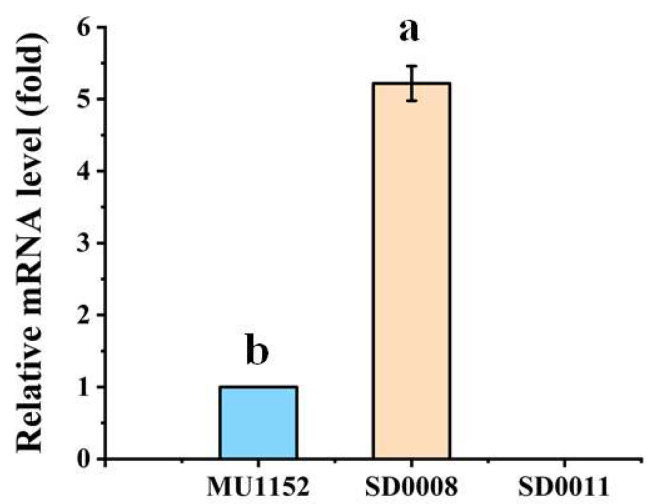
Transcription levels of the *ada* gene in the overexpressing strain SD0008, knockout strain SD0011, and the wide-type strain MU1152. Error bars represent the standard deviations. Different letters indicate significant differences, *p* < 0.05. For each group, three biological repetitions were used.

The relative mRNA levels of *ada* in the control MU1152, *ada*-overexpressing strain SD0008 and *ada*-knockout strain SD0011 were measured by qRT-PCR. Compared to the control strain, the transcription level of *ada* in the *ada*-overexpressing strain (SD0008) increased 5.2-fold at 24 h, which confirmed the *ada* gene was successfully overexpressed. However, the *ada* gene was deleted in SD0011, according to a marginal expression level of *ada* as shown in Figure 3.

### 2.4. Ada Regulated Nitrogen Metabolism and Affected Cell Growth and Lipid Accumulation

To investigate the role of ADA in the lipid biosynthesis of *M. circinelloides*, cell growth and lipid accumulation in SD0008 (*ada*-overexpressing strain) and SD0011(*ada*-knockout strain) were analyzed during 96 h of cultivation in K&R medium, supplemented with leucine.

**Figure 4:**
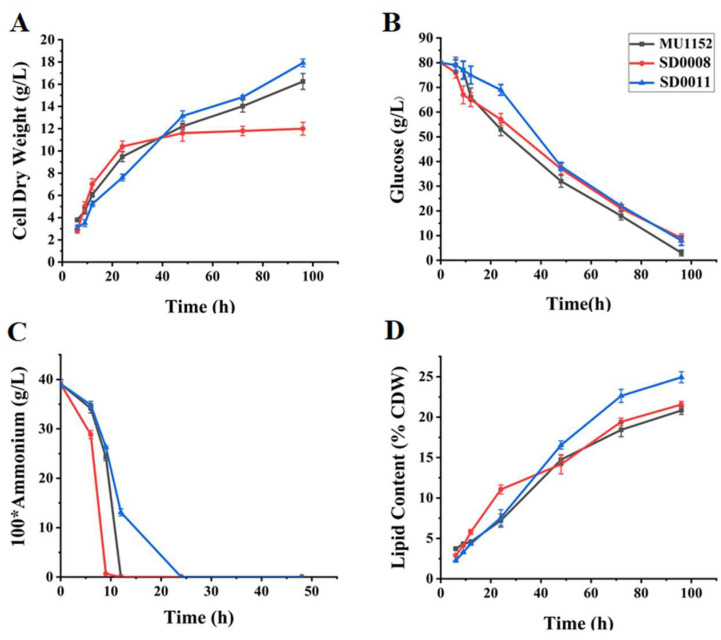
Cell dry weight (**A**), residual glucose (**B**), ammonium (**C**), and lipid accumulation (**D**) of *ada*-overexpressing and *ada*-knockout strains cultured in 1 L K&R medium were measured. The values are means ± standard deviations (bars) of the three independent experiments.

As shown in Figure 4A, the cell growth patterns of three strains were affected by the *ada* gene expression level. Overexpression of *ada* increased nitrogen assimilation, cell growth, and the lipid accumulation of the fungus before 24 h of cultivation (from 7.20% in the control to 11.05% in SD0008) (Figure 4D). After that, cell proliferation of the *ada* overexpressing strain was inhibited and the cell dry weight was much less than the control, while the lipid accumulation rate (0.13 g/h) was lower than that in the control (0.31 g/h). Thus, the final cell lipid content (at 96 h) was nearly the same as that of the control strain. In contrast, *ada* disruption decreased nitrogen assimilation and cell growth before 24 h of cultivation, while at the later stage, lipid-free CDW increased by about 4.51% (Figure S2) and the lipid accumulation was increased by 20% compared to the control. Although it is not clear how the *ada* gene regulated the growth and lipid biosynthesis in the transformants, the results indicated that *ada* altered nitrogen depletion to face the cellular stress.

### 2.5. Impacts of Ada Gene Manipulation on the Expression Levels of the Key Genes for Fatty Acid Biosynthesis

Among the essential enzymes involved in lipid biosynthesis in *M. circinelloides*, ATP-citrate lyase (ACL) was encoded by *acl* (gene ID: scaffold00037.10), acetyl-CoA carboxylase (ACC) was encoded by *acc1* (gene ID: scaffold00021.30) and *acc2* (gene ID: scaffold00023.50), the cytosolic malic acid enzyme (ME) was encoded by *cme1* (gene ID: scaffold00036.12) and *cme2* (gene ID: scaffold00049.37), and fatty acid synthase was encoded by *fas1* (gene ID: scaffold00002.57) and *fas2* (gene ID: scaffold00111.12). In addition, as the negative regulator for lipid accumulation, AMP-activated protein kinase (AMPK) contains three subunits; its α subunit was encoded by *ampk α1* (gene ID: scaffold00144.16) and *ampk α2* (gene ID: scaffold00046.26), and its β subunit was encoded by *ampk β* (gene ID: scaffold00011.47), respectively. As the manipulation of the ADA expression level made a difference in the lipogenesis of *M. circinelloides*, the effects of *ada* overexpression and deletion on the expression of the pivotal genes in this fungus mentioned above were investigated by qRT-PCR at 24 h, when the nitrogen was exhausted in the fermentation medium.

**Figure 5:**
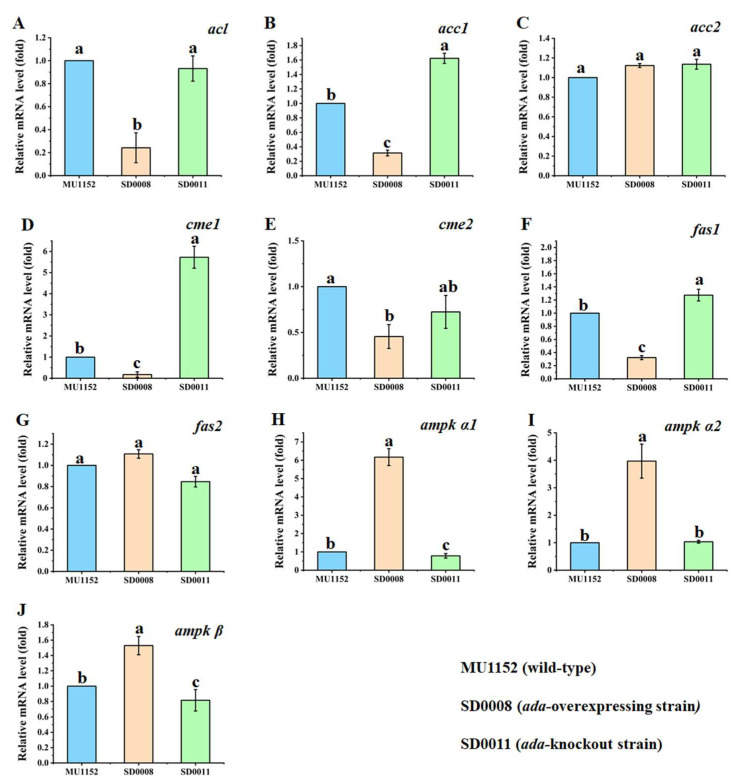
Expression profiles of key genes in *ada*-overexpressing and *ada*-knockout strains at 24 h. (**A**) *acl*, ATP-citrate lyase; (**B**) *acc1*, (**C**) *acc2*, acetyl-CoA carboxylase; (**D**) *cme1*, (**E**) *cme2,* cytosolic malic acid enzyme; (**F**) *fas1*, (**G**) *fas2,* fatty acid synthase; and (**H**) *ampk* *α1*, (**I**) *ampk* *α2,* (**J**) *ampk* *β,* AMP-activated protein kinase. Data were collected from three biological replicates and showed significant differences (*p* < 0.05) when they do not share common superscripts.

Our results showed that the relative expression levels of *acc1* and *fas1* were slightly increased when *ada* was deleted (Figure 5B,F). In contrast, *acl*, *acc1,* and *fas1* expression levels were significantly downregulated in the *ada*-overexpressed mutant (Figure 5A,B,F). Meanwhile, the transcriptional levels of *acc2* and *fas2* of the recombinant strains had no differences compared with the control (Figure 5C,G). Figure 5D,E show the lessened transcription levels of both *cme1* and *cme2* in SD0008 compared with those of the control strain, while the *cme1* gene was considerably upregulated in the *ada*-knockout strain. As a negative regulator of lipid biosynthesis, the three genes encoding AMPK were extremely upregulated in the *ada*-overexpressed mutant and the relative mRNA levels of *ampk α1* and *ampk β* were decreased slightly in the *ada*-knockout mutant (Figure 5H–J). The qRT-PCR results suggested that the manipulation of *ada* can regulate the expression of *acc1*, *fas1*, and *ampk*, and therefore contribute to the alteration of lipid metabolism in *M. circinelloides*.

## 3. Discussion

Lipid accumulation can be triggered when nitrogen is depleted in the growth medium, which is a unique process in the cytoplasm of oleaginous microorganisms. As nitrogen is one of the major constituents of many cellular compounds, the cells stagnated the biosynthesis of proteins and nucleic acids after nitrogen deficiency which caused the excessive carbon flux flowed to fatty acid biosynthesis [31,32]. Thus, ADA, as a crucial enzyme involved in the biosynthesis and regulation of nucleic acids and protein [20,33], should have an important role in the switching of protein/nucleic acid biosynthesis to the lipid accumulation process [19]. However, only a few studies have investigated the function of ADA in autoimmune diseases and type 2 diabetes mellitus [25,34,35], and no significant work has elucidated its effect on lipid accumulation. In this study, we identified an ADA encoding gene in oleaginous fungus, *M. circinelloides* WJ11, which contained an ADA_AMPD domain (cd00443). The phylogenetic tree for ADA in WJ11 and the homologous proteins from other species revealed that ADA was conserved in animals, plants, and fungi (Figure 1), which showed catalytic activity in adenosine deamination. In addition, the previous work illustrated that the depletion of adenosine by ADA enhanced insulin sensitivity, which inhibited lipolysis in the isolated adipose tissue of humans and rats [36,37]. Therefore, ADA connects nitrogen metabolism and lipid biosynthesis in an unknown regulating mechanism. 

To unravel the novel role of ADA in governing fatty acid synthesis in oleaginous fungi, we constructed the *ada* gene overexpression and knockout strains by genetic strategies (Figure 2). Growth analysis revealed that the overexpression of *ada* resulted in the enhanced cell growth and efficiency of ammonium utilization before 24 h compared to the control (Figure 3A,C), which resulted in an early nitrogen exhaustion in SD0008 (Figures S3 and S4). Subsequently, the lipid accumulation was triggered earlier in SD0008, which led to a higher lipid production in the gene overexpressing transformant in the early stage (Figure 3A,D). However, because of the early nitrogen exhaustion, the cell growth was inhibited after 24 h during the fermentation process in SD0008. A biochemical hypothesis has been postulated that fungal mycelia continue to uptake glucose even though nitrogen deficiency limited cell growth, thus obliging the organism to accumulate surplus carbon as lipids [38]. Nevertheless, there was no evidence that the inhibited cell proliferation at the later stage caused by *ada* overexpression has an influence on the final lipid production. However, disruption of *ada* significantly retarded the absorption of nitrogen and showed a negative effect on the cell growth in the presence of nitrogen compared to the control, but at the later stage of growth (after 24 h), both growth (13.45 g/L of lipid-free CDW in SD0011 and 12.87 g/L of lipid-free CDW in the control) and lipid production (24.93% in SD0011 and 20.82% in the control) were enhanced (Figure 3D), suggesting that the absence of ADA improved carbon flux from cell growth to lipid accumulation, due to its role in the regulation of nitrogen metabolism. Similarly, the adenosine concentration in the extracellular space was regulated and adenosine’s action on its membrane receptors was modified by ADA in human [39,40]. Moreover, the delay in cell growth before 24 h may result from events associated with the accumulation of extracellular adenosine, which inhibited cell proliferation, and resulted in the further deceleration of the nitrogen uptake [41,42]. Additionally, Hoshinoa et al. revealed that the removal of endogenous adenosine by ADA resulted in an immediate rise in lipolytic activity [43]. Therefore, the stock of adenosine could be a possible cause for the increased lipid production in the *ada*-knockout strain, since adenosine has been considered to be a major endogenous antilipolytic factor [44], regulating the balance between lipolysis and lipogenesis [45,46].

To further investigate the specific role of ADA in fatty acid biosynthesis, we conducted the qRT-PCR experiment to test the expression levels of lipid metabolism-related genes in the transformants. In most oleaginous organisms, ACL and ME are proposed to provide, respectively, acetyl-CoA and NADPH for fatty acid (FA) synthesis [47,48]. The acl gene from *M. musculus* overexpressed in oleaginous yeast, *Y. lipolytica,* enhanced the lipid accumulation from 7.3% to 23.1% of CDW, whereas the inactivation of *ACL1* of *Y. lipolytica* decreased FA synthesis by 60 to 80% [49,50]. Additionally, the overexpression of ME genes from *M. circinelloides* improved lipid accumulation by 2.5- and 2-fold in *M. circinelloides* and *Rhodotorula glutinis*, separately [13,51]. Therefore, compared with the control, the similar lipid production in the *ada*-overexpressed strain was related to the insufficient precursors and NADPH, which consistent with the declined mRNA levels of *acl* and *cme*, as displayed in Figure 5A,D,E. The transcriptional results of *acc1* and *fas1* (Figure 5B,F) showed significant increments in the knockout mutant compared to the other two strains. As the first and last step of palmitic acid biosynthesis, the substantial function of ACC and FAS in regulating lipid biosynthesis has been widely studied in various organisms. It has been documented that *acc* overexpression in the non-oleaginous yeast and bacteria, as well as in some plants, showed an overall enhancement in fatty acid production [52,53,54,55]. Similarly, the expression of FAS systems in *Saccharomyces cerevisiae* triggered short-chain fatty acid production, whereas the degradation of *FASs* in parasitic lifecycle-based insect species led to the loss of lipogenesis [56,57]. Thus, the evidence suggested that increased expression of *acc1* and *fas1* genes is likely to result in the promotion of the conversion of the precursors for fatty acid synthesis, which ultimately leads to more lipid production in the *ada*-knockout strain [58,59]. AMPK, acted as a negative regulator of lipid biosynthesis in oleaginous fungi [60,61], was also chosen to check the exchanged expression levels affected by *ada* manipulation. It is noteworthy that a significant upregulation of the AMPK subunit genes (*α1*, *α2*, and *β*) in the *ada*-overexpressed mutant was observed compared to that of the control (Figure 5E–G). The downregulation of AMPK genes observed in the *ada* disruption mutant could also be consistent with an impaired ability for fatty acid exploitation, that may be an explanation for the increased lipid production [62,63].

One of the unexpected findings from our study was that ADA was involved in the nitrogen metabolism of *M. circinelloides*. However, the lipid yield generated by the *ada*-knockout mutant was higher, indicating that the activation of ADA revealed the negative effects in motivating the lipid production. Considering the ability to modulate signaling metabolites in cells, the ADA in *M. circinelloides* may also regulate lipid metabolism through the signal molecules. Recently, research showed that ADA also increased the levels of the second messenger, cyclic AMP (cAMP), which leads to the activation of cAMP-dependent protein kinase A (PKA) and stimulates lipolysis together with other proteins [64,65]. Therefore, studies should be continued to perform a deeper analysis and to fully understand the function and regulation mechanisms of ADA in oleaginous organisms.

## 4. Materials and Methods

### 4.1. Strains, Transformation, and Fermentation Conditions

The *Escherichia coli* DH5α was used for cloning and plasmid construction and propagation, and was grown in Luria–Bertani (LB) medium at 37 °C, with shaking at 200 rpm, supplemented with ampicillin or kanamycin (100 mg/L) for plasmid maintenance [66]. Strain MU760, a leucine and uracil auxotroph of *M. circinelloides* WJ11, was used as the recipient strain in all the transformation experiments. Cultures were grown at 28 °C in YNB, YPG, or MMC medium, which were adjusted to pH 4.5 or 3.2 for mycelial or colonial growth, separately, and supplemented with uracil (200 μg/mL) or leucine (600 μg/mL) when required [18]. Transformation and selection procedures were carried out as previously described [67].

The fungal spores (~10^5^ to 10^6^) of each *M. circinelloides* strain were inoculated into 100 mL of Kendrick and Ratledge (K&R) medium in 500 mL flasks equipped with baffles and cultured in a rotating shaker at 130 rpm, at 28 °C for 24 h, and the resultant seed cultures were used for inoculation at 10% (*v*/*v*) into a 1.5-L bioreactor [68]. These fermenters were operated at 28 °C and stirred at 700 rpm, with aeration at 2.0 vvm, and pH controlled at 6.0 by 2 mol/L NaOH. The high nitrogen medium was the same as the modified K&R medium, except for ammonium tartrate at 10 g/L.

### 4.2. Identification and Bioinformatics Analysis of ADA Gene in M. circinelloides

Based on the genome annotation of *M. circinelloides* WJ11, a putative ADA gene was retrieved according to the gene information and the presence of the conserved domains of ADA proteins from other organisms. The phylogenetic tree was constructed by using the MEGA 6.0 program, based on the sequences of ADA obtained from the NCBI database using systematic BLAST searches (www.ncbi.nlm.nih.gov/BLAST, accessed on 10 September 2015). The molecular weight, protein isoelectric point, instability index, aliphatic index, and tertiary structure were analyzed by using ProtParam (https://web.expasy.org/protparam/, accessed on 10 September 2015), CFSSP (https://www.biogem.org/tool/choufasman/index.php, accessed on 10 September 2015), SOPMA (https://npsa-prabi.ibcp.fr/cgi-bin/npsa_automat.pl?page=npsa_sopma.html, accessed on 10 September 2015), and SWISS-MODEL (https://swissmodel.expasy.org, accessed on 10 September 2015).

### 4.3. Plasmid Construction

The plasmid pCRC8 was constructed for overexpressed *ada* gene in *M. circinelloides* by the modification of pMAT2075 [69], harboring a construction contained in the *ada* gene under the promoter of *zrt1*, as well as the *pyrF* gene of *M. circinelloides* as a selectable marker, which was surrounded by 1 kb up- and downstream sequences of the *carRP* gene to allow its chromosomal integration by homologous recombination. The *ada* fragment was obtained by PCR amplification from the genome of *M. circinelloides* WJ11 using the primers ada-F/ada-R, which contained 25 bp homologous sequences on both sides of linearized pMAT2075, cut by *Nhe I* restriction endonuclease. The PCR fragment was cloned into linearized pMAT2075 to generate plasmid pCRC8 by using the one-step cloning kit (Takara). The primer sequences can be found in Supplementary Table S1.

Plasmid pCRC43 was constructed to disrupt the *ada* gene that comprised the *M. circinelloides pyrF* gene, flanked by 1 kb of the up- and downstream regions of the *ada* gene, according to the previously described method [18]. The up- and downstream fragments of the *ada* gene were obtained by PCR amplification with the primer pairs, adaup-NotΙ-SmaΙ-F/adaup-R and adadown-F/adadown-NotΙ-SmaI-R, separately. Then, the *pyrF* was amplified using the primer pair pyrF-F/pyrF-R. These three fragments were joined using fusion PCR with the primer pair adaup-NotΙ-SmaΙ-F/adadown-NotΙ-SmaI-R, and the PCR product was cloned into pUC18 after digested with *Not I* and *Sma I*.

### 4.4. Biochemical Analysis of the Fermentation Process

The biomass of the fungal strains was filtered using a Buchner funnel and washed thrice with distilled water to remove the medium excess, and frozen overnight at −80 °C. Then, the frozen biomass was lyophilized and determined gravimetrically. The glucose oxidase Perid-test kit (Rongsheng) was used to determine the glucose concentration in the medium, and ammonium was calculated by the indophenol method [70].

### 4.5. Determination of Lipid Accumulation in Transformants

The lipids were extracted from the mycelia as previously described by Folch et al., with minor modifications [71]. Approximately 15 mg of dry mycelia was mixed with the chloroform/methanol (2:1, *v*/*v*), and the pentadecanoic acid (15:0) was used as the internal standard. Methylation was performed with 10% HCl/methanol (*w*/*w*) and the fatty acid methyl esters (FAMEs) were separated with *n*-hexane (HPLC grade). Finally, the FAMEs were analyzed by gas chromatography (GC) equipped with a column: 30 m × 0.32 mm, 0.25 μm (DB-Waxetr). The program was: 120 °C for 3 min, ramp to 200 °C at 5 °C/min, then ramp to 220 °C at 4 °C/min, and hold for 2 min. Finally, the lipid content was calculated from the data determined by GC.

### 4.6. RNA Isolation and Gene Expression Analysis by qRT-PCR

The qRT-PCR was subsequently carried out to quantify the levels of genes expression. The total RNA was extracted from the fungal mycelium after being grown in a 1 L fermenter for 24 h. The qRT-PCR was performed using specific primers (Supplementary Materials Table S1) and the SYBR Green Realtime PCR Master Mix kit (Roche). The *actin* gene of *M. circinelloides* was served as the housekeeping gene. All the data were analyzed through relative quantification for qRT-PCR (2^−ΔΔCt^).

### 4.7. Statistical Analysis

All the experiments were performed in triplicate and data were presented as means ± S.D. The Student’s *t* test of IBM SPSS Statistics 22 was used for statistical analysis of the results, and *p* < 0.05 was considered as a significant different.

## 5. Conclusions

In this study, the results provided evidence for the important roles of ADA in lipid accumulation and nitrogen metabolism in *M. circinelloides* WJ11. Analysis of overexpression and deletion strains for the ADA gene revealed that ADA, as a regulator for lipid biosynthesis, regulated the expression levels of genes encoding the key enzymes of lipolysis and lipogenesis and, therefore, affected the redistribution of carbon flux. Specifically, we showed that the genetic modification of ADA in *M. circinelloides* altered the capacity of nitrogen consumption and lipid biosynthesis. Nevertheless, further investigation is needed regarding how ADA influences nitrogen metabolism and achieves a high lipid yield in a shorter fermentation time.

## Data Availability

Not applicable.

## Supplementary Materials

The following supporting information can be downloaded at: https://www.mdpi.com/article/10.3390/jof8080774/s1, Figure S1: The predicted three-dimensional (3D) structure of ADA in *M. circinelloides*; Figure S2: Lipid-free cell dry weight (CDW); Figure S3: Lipid-free CDW/Glucose; Figure S4: Lipid-free CDW/Ammonium; Table S1: List of primers used in this study; Table S2: Cell growth rate of mutants and the control strain.
