# Supplementary material for: Identification and Functional Characterization of Adenosine Deaminase in Mucor circinelloides: A Novel Potential Regulator of Nitrogen Utilization and Lipid Biosynthesis
Source: J Fungi (Basel). 2022 Jul 26;8(8):774. doi: 10.3390/jof8080774 (PMC9332508; doi:10.3390/jof8080774)
Supplement: Supplementary file 1 [file jof-08-00774-s001.zip › jof-1815866-SI.pdf]

Table S1. List of primers used in this study

Primer	Sequence
ada-F	TAACTAAATTACGTAGCTAGCATGACTGTCCCAATTTTGG
ada-R	CTGCCTCGAGGCTAGCTTAGAACTGTGATTGGTTCTCTG
adaup-NotI-SmaI-F	TGACTCCCGGGGCCTGCGGCCGCGTCCAAATTCTTCATGGACAG
adaup-R	GCATGTATTACCTGCAGTAACAAGACCTGGAAGCCGATGTGAAGAA
pyrF-F	ATCTTCTTCACATCGGCTTCCAGGTCTTGTTACTGCAGGTAATACATGC
pyrF-R	GATGCGATTTCCTAGCTTAGTAGCCGTTGCTAAATCCAGATTTCACATGG
adadown-F	CCATGTGAAATCTGGATTTAGCAACGGCTACTAAGCTAGGAAATCGCATC
adadown-NotI-SmaI-R	TAGTTCCCGGGCGGCCGCCTAGATGCATCGTCTAATGCTCC
ada-overF1	GATAAGCATAAACCAGATCTGC
ada-overR1	GTATCTGACATAGTCGAGCTTG
ada-outF2	CCTCAGGTGTCTTTGCATC
ada-outR2	ATGCTGGATTGCGATCCAAC
actin-F	GATGAAGCCCAATCCAAGAGAGGT
actin-R	TCTTCTCACGGTTGGACTTGGG
acc1-RT-F	GATATGCATTCCCGTGTTTCCG
acc1-RT-R	ACCGAGTAATTCATCAGCTGGG
acc2-RT-F	GCTGAAGCTGGTGGTATTGCC
acc2-RT-R	GATACGAGCATTAGGCCACATC
fas1-RT-F	ACTGGTATCCGTTTCATTGAGCCTG
fas1-RT-R	AAATGTCTGCCTTTTCTCCCTGTTGA
fas2-RT-F	GGTCTTCGTTTCATTGACCCCACTG
fas2-RT-R	GACATCAGCCTTGTCACCTTGTTGG
ampkα1-RT-F	GGCATGGAGTGGCGAACATTG
ampkα1-RT-R	TTGTGGTTGTGGTGCATCGC
ampkα2-RT-F	GGAGAACGTGCTCATTGATAACAC
ampkα2-RT-R	CCACAAATCGACAGGAGGTC
ampkβ-RT-F	GTAACGTCAAATGCGCCAAC
ampkβ-RT-R	CTGCACTGAAATCATGCGTGC

Table S2. Cell growth rate of mutants and the control strain

Time	MU1152	SD0008	SD0010
6h-9h	0.27±0.02^{b}	0.73±0.02^{a}	0.14±0.02^{c}
9h-12h	0.49±0.03^{c}	0.67±0.04^{a}	0.57±0.03^{b}
12h-24h	0.29±0.02^{a}	0.28±0.05^{a}	0.20±0.03^{b}
24h-48h	0.11±0.05^{b}	0.05±0.01^{c}	0.23±0.03^{a}
48h-72h	0.08±0.03^{a}	0.01±0.01^{b}	0.07±0.02^{a}
72h-96h	0.09±0.04^{a}	0.01±0.01^{b}	0.13±0.02^{a}

The values are the means ± standard deviations of the three independent experiments and significantly different from each other ($p < 0.05$) when they do not share common superscripts.

Figure S1. The predicted three-dimensional (3D) structure of ADA in *M. circinelloides*

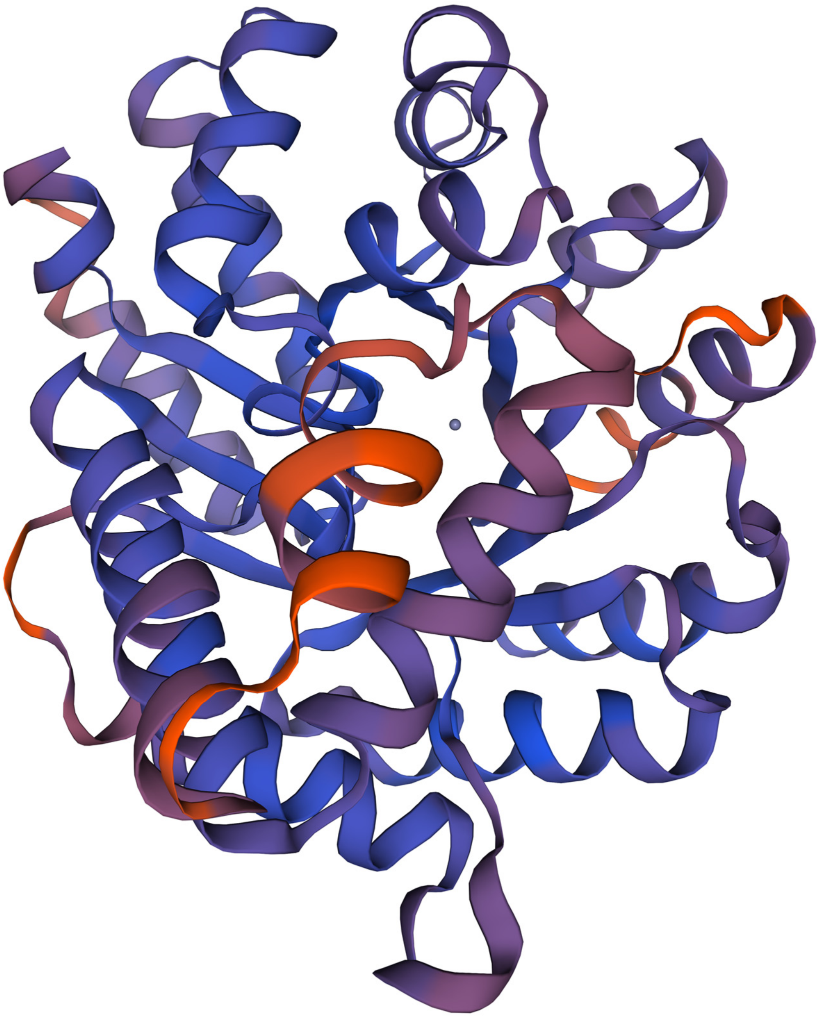

Figure S2. Lipid-free cell dry weight (CDW)

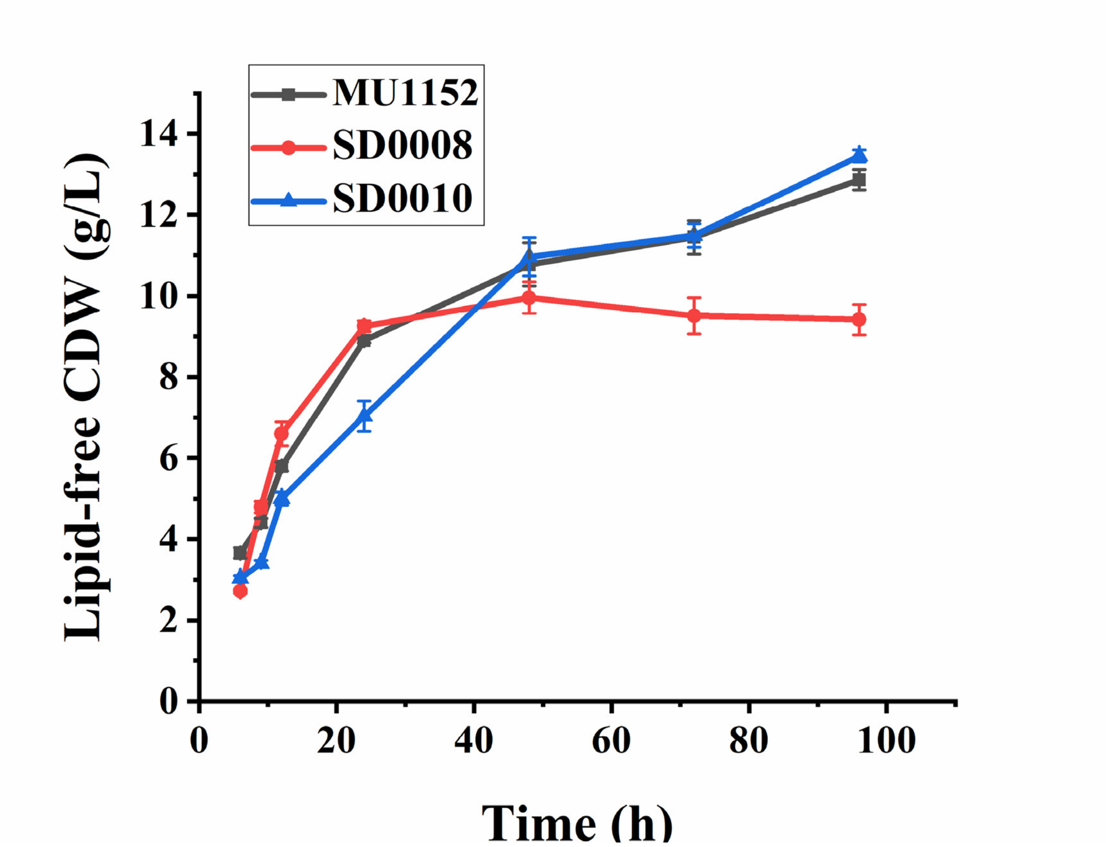

Figure S3. Lipid-free CDW/Glucose

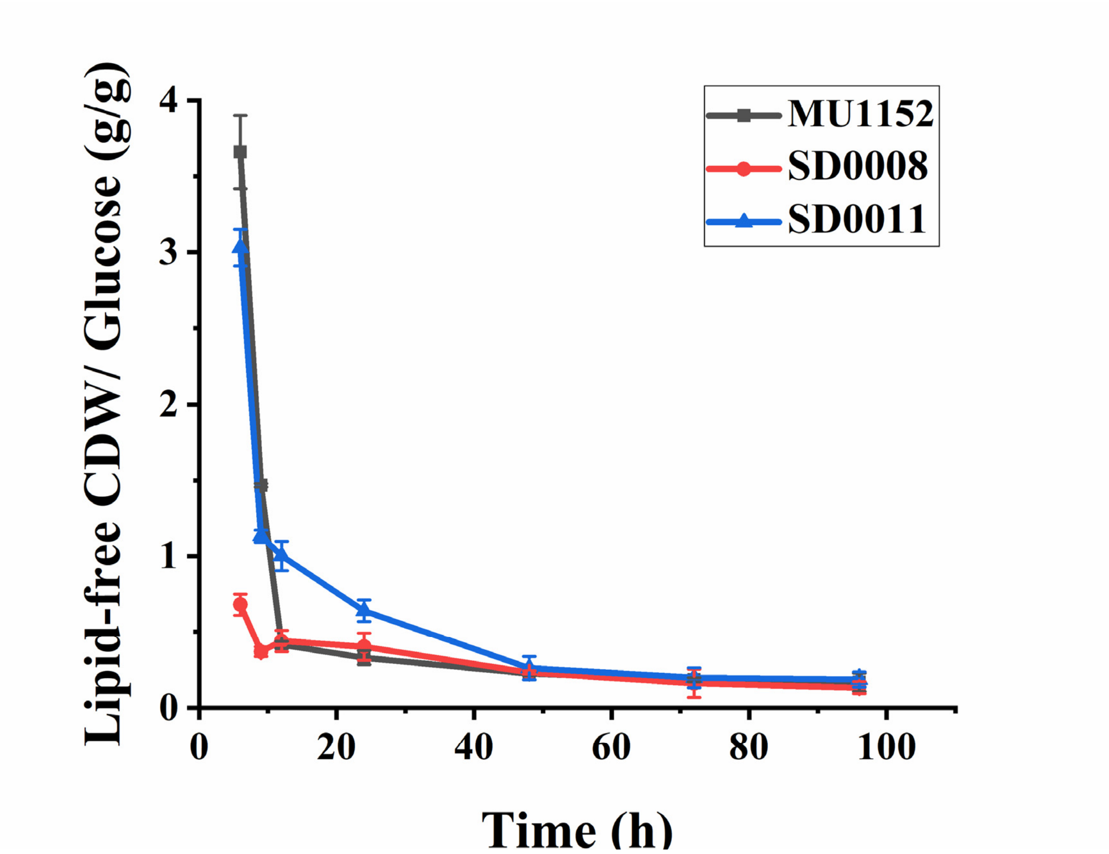

Figure S4. Lipid-free CDW/Ammonium

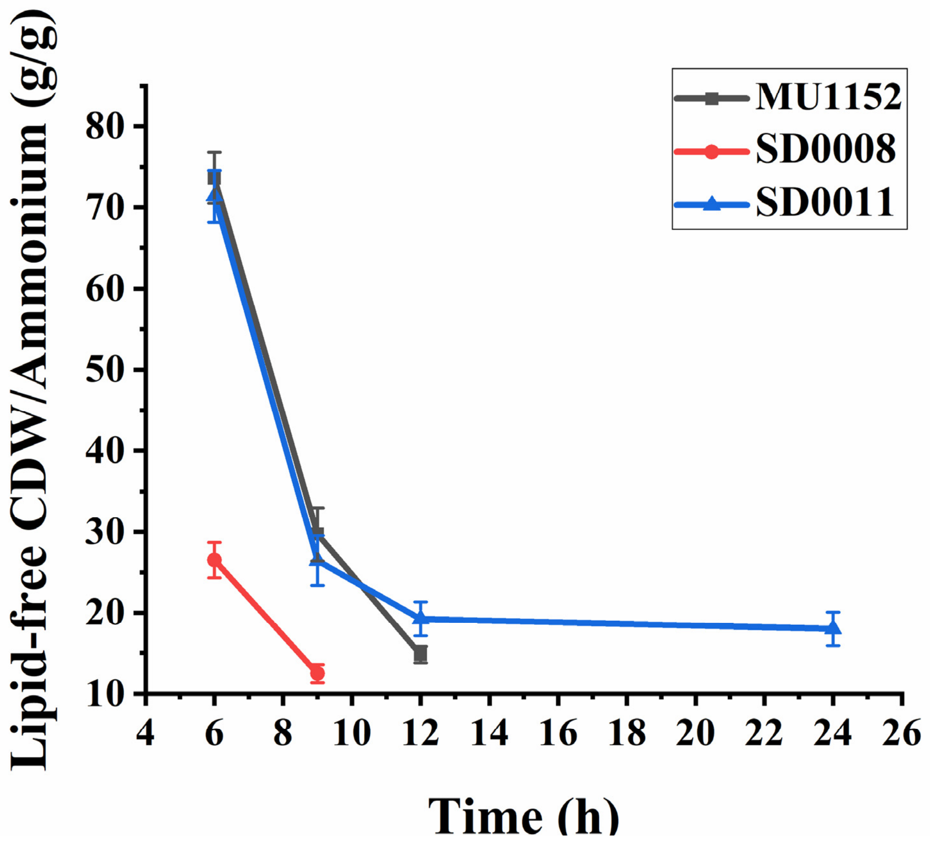

MU1152
SD0008
SD0011
Lipid-free CDW/Ammonium (g/g)
80
70
60
50
40
30
20
10
4
6
8
10
12
14
16
18
20
22
24
26
Time (h)
